# Maternal caffeine intake during pregnancy and the risk of delivering a small for gestational age baby: Kuopio Birth Cohort

**DOI:** 10.1007/s00404-024-07538-7

**Published:** 2024-05-20

**Authors:** Anni Kukkonen, Sari Hantunen, Ari Voutilainen, Anu Ruusunen, Katri Backman, Pirkka V. Kirjavainen, Maija Ylilauri, Raimo Voutilainen, Markku Pasanen, Leea Keski-Nisula

**Affiliations:** 1 Institute of Public Health and Clinical Nutrition, University of Eastern Finland, Kuopio, Finland; 2 Department of Pediatrics, Kuopio University Hospital, Kuopio, Finland; 3 Department of Psychiatry, Kuopio University Hospital, Kuopio, Finland; 4 School of Medicine, IMPACT—the Institute for Mental and Physical Health and Clinical Translation, Barwon Health, Deakin University, Geelong, Australia; 5 Institute of Clinical Medicine, School of Medicine, University of Eastern Finland, Kuopio, Finland; 6 Environmental Health Unit, National Institute for Health and Welfare, Kuopio, Finland; 7 School of Pharmacy, University of Eastern Finland, Kuopio, Finland; 8 Department of Obstetrics and Gynecology, Kuopio University Hospital, Kuopio, Finland

**Keywords:** Caffeine, Coffee, Maternal diet, Small for gestational age, Neonatal health

## Abstract

**Purpose:**

High caffeine intake during pregnancy is associated with restricted fetal growth. We aimed to evaluate the association between maternal caffeine intake during early and late pregnancy and the risk of delivering a small for gestational age (SGA) baby.

**Methods:**

Kuopio Birth Cohort (KuBiCo) is a prospective cohort study including women whose pregnancies and deliveries were treated at the prenatal clinics in outpatient healthcare centers and in Kuopio University Hospital, Finland. Maternal diet and caffeine intake during the first (*n* = 2007) and third (*n* = 4362) trimester of pregnancy were assessed using a 160-item food frequency questionnaire (2013–2022). SGA was defined as birth weight corrected for gestational age below  − 2 standard deviations from the mean, according to the sex-specific Finnish fetal growth curves.

**Results:**

Altogether in 32 and 38% (1st and 3rd trimester) of all women and in 44 and 52% of coffee drinkers, caffeine intake exceeded the recommendation for caffeine intake ($\le$200 mg/day) during pregnancy. The women with moderate (51–200 mg/day) (aOR 1.87; 95% CI: 1.16–3.02) and high (> 200 mg/day) (aOR 1.51; 95% CI: 1.08–2.10) caffeine intake during the first trimester were in the highest risk of having an SGA newborn. Caffeine intake in the third trimester of pregnancy was not associated with SGA.

**Conclusions:**

Moderate and high caffeine intake during early pregnancy is associated with SGA. As the results suggest that even moderate caffeine intake during the first trimester may increase the risk of SGA, the intake within recommendation limits does not necessarily appear to be safe for pregnant women and their newborns.

## What does this study add to the clinical work

**Table:** 

Special attention should be paid to consumption of caffeine in early pregnancy and when planning for pregnancy.	

## Introduction

Caffeine is a widely used central nervous system stimulant among pregnant women. It presents in many foods and drinks, and coffee and tea are the major sources of caffeine in the maternal diet worldwide [1–3]. According to European Food and Safety Authority (EFSA) [4], pregnant women should limit daily caffeine intake to 200 mg per day which equals to two small cups (150 ml) or one mug (300 ml) of coffee. However, in our recent study, we found that more than 40% of pregnant women in our Finnish cohort consumed more caffeine than recommended [5].

The EFSA recommendation is based on the prospective cohort studies, which have shown a dose-dependent association between maternal caffeine intake and weight-related pregnancy outcomes, such as low birth weight, small for gestational age (SGA) and fetal growth restriction (FGR) [1, 6]. Several recently published observational studies and meta-analyses have also confirmed that higher maternal caffeine intake is associated with an increased risk of low birth weight [7–9] and poorer fetal growth, such as lower birth length, and head and/or thigh circumference [3, 9]. Even the use of caffeine within recommendations has been related to reduced fetal growth [9]. Although higher maternal caffeine intake is associated with an increased risk for diverse pregnancy outcomes such as miscarriage and stillbirth [10–13], according to the meta-analyses, the higher intake of caffeine does not appear to increase the risk of preterm birth [10, 14, 15].

SGA is typically defined as children born below the 2.5th–10th percentile of the reference birthweight by sex and gestational weeks in a region [16–18]. Unlike preterm birth (delivery  < 37 weeks), the gestational age at birth of SGA newborns may therefore be normal, and on the other hand, some of them may be genetically small but healthy [19]. Globally, the prevalence of preterm SGA was estimated to be 1.1% and term SGA 16.3% [20], but the prevalence varies across different countries in relations to the populations and definition criteria. SGA is strongly related to neonatal health problems [21], the need for neonatal intensive care [22, 23], and neonatal morbidity and mortality [24]. In addition to the acute health problems of newborns, insufficient fetal growth may increase the risk of diabetes and cardiovascular diseases in adulthood [25–27]. Besides maternal caffeine intake, also maternal factors like poor nutritional status, infections, hypertension, smoking, alcohol and drug use, poor placental function and various fetal factors have been associated with the development of SGA [19, 28].

Caffeine is absorbed rapidly and it crosses the placenta freely [29, 30]. Since the fetus and placenta lack the major xenobiotic metabolizing enzymes (e.g. cytochrome P450 1A2; CYP1A2) for caffeine metabolism, it relies on the maternal metabolic capacity. The maternal enzyme function decreases during pregnancy and the half-life of caffeine increases from 5 to 18 h from the first to the third trimester [31]. Due to these metabolic changes, fetal exposure to caffeine increases and it accumulates to fetal tissues [32]. High caffeine levels in maternal blood are accompanied by higher cyclic adenosine monophosphate (cAMP), greater inhibition of phosphodiesterase (PDE) [33], and higher epinephrine levels [29] leading to an increased risk of intrauterine fetal asphyxia, pregnancy loss and restricted fetal growth. Genetic polymorphisms may influence the potential effects of caffeine and its metabolites on perinatal outcomes in different races [34].

Most of previous studies on maternal caffeine intake have focused on the anthropometry of newborns and gestational length. In this study, we prospectively examined the association between the maternal first and third trimester caffeine intake measured by a food frequency questionnaire (FFQ), SGA birth and neonatal health in the Kuopio Birth Cohort Study (KuBiCo) in Finland.

## Methods

### Study participants

In KuBiCo, pregnant women were recruited (majority,  > 90% during the first trimester) by personnel at the prenatal clinics of outpatient healthcare centers in Northern Savo, Finland. The only inclusion criteria in the study were that the participants were expected to give birth after 22nd gestational week in Kuopio University Hospital (KUH) and to be proficient in Finnish.

During the first and third trimesters of pregnancy, three questionnaires were filled out by the study participants (Environmental exposure questionnaires, Food frequency questionnaires (FFQ1 and FFQ2) and Edinburgh Postnatal Depression Scale questionnaires). In addition to these, all pregnant women filled out various questionnaires evaluating the background risk for ongoing pregnancy in Kuopio University Hospital’s Pikku-Haikara system intended for pregnant women. By agreeing to the KuBiCo study, the women give permission for the use of all those and hospital data related to pregnancy and childbirth in the KuBiCo study.

In this study, we included those who had given birth in KUH and whose dietary data were available. In total, 2007 (the first trimester) and 4362 (the third trimester) pregnant women, who had completed the FFQ between March 2013 and December 2022, were included in the study. Of these women, 1262 had completed both the first and third trimester FFQ. All women gave informed consent during recruitment. The study data were collected year-round with standardized, supervised methods according to the study protocol and it represent Finnish pregnant women well [35]. The overall participation in KuBiCo during 2013–2022 (7944 women) has been 35.2% of all parturients giving birth in KUH.

### Maternal dietary assessment

Maternal diet was estimated using an FFQ filled by the participants during the first (up to 13rd week) and the third trimester of gestation (from 28th gestation week to delivery). The FFQ was designed to cover the whole diet during the preceding three months. Average daily nutrient and energy intakes were calculated using the Finnish national food composition (Fineli®) database in February 2023.

The FFQ used was a slightly modified version from that validated in the Kuopio Breast Cancer study [36]. To reflect the increased selection of food items available, for example information on the use of energy drinks and bars, were collected in the modified FFQ. In the KuBiCo, the participants completed the web-based FFQ which also produced automatized personal feedback to all study participants within 24 h after they returned the online FFQ.

The FFQ comprises a food list of 160 food and drink items with 9 frequency response options, ranging from ‘never’ to ‘6 or more times per day’. The portion sizes were fixed and specified with natural units (e.g., mug, glass, slice) or grams. The average daily intake of listed food items and nutrient intakes were calculated by multiplying the frequency of the food intake by the portion size (grams). The output provided more than 60 nutrients and 100 food groups. The caffeine intake was estimated by summarizing the daily intake of coffee (caffeine 70 mg/100 ml), cola drinks (20 mg/100 ml), tea (15 mg/100 ml), energy drinks (32 mg/100 ml), cocoa (3 mg/100 ml), and chocolate (30 mg/100 g).

### Maternal and neonatal health examinations

In this study, the following descriptive characteristics were collected from the KUH birth registry: maternal age, body mass index (BMI; kg/m^2^) in the first trimester, weight change during pregnancy, smoking during pregnancy, the duration of education, relationship status, parity, the mode of delivery, and maternal diseases and complications. Also, newborn sex, the gestational age at birth, birth weight, head circumference, Apgar Scores (at 5 min), the duration of hospital stay after birth, the need of neonatal intensive care, and congenital anomaly (ICD-10, Q Codes) at birth, were recorded. According to WHO [37], low birth weight was defined as birth weight  < 2500 g, and preterm birth was defined as delivery before 37 weeks of gestation. Macrosomia was classified as birth weight  > 4500 g [38]. Further, we divided the birth weights into two subgroups according to birth weight for gestational age: SGA and large for gestational age (LGA). SGA and LGA were defined as birth weights corrected for gestational age beyond  ± 2 standard deviations (SD) from the mean, according to the sex-specific Finnish fetal growth curves [39]. Maternal hypertension and pre-eclampsia were defined according to ICD-10 codes (O10, O11, O13, O14, O15 and O16).

### Data analysis

The statistical analyses were conducted using IBM SPSS Statistics version 25 (SPSS Inc., Chicago, IL, USA). *P*-values  < 0.05 were considered as significant. Caffeine intake was classified into three categories: low $\le$50 mg/d, moderate 51–200 mg/d and high caffeine intake  > 200 mg/d. Caffeine and caffeine containing food intakes were expressed as the mean values with SD and the median values with interquartile range (IQR). Differences between the first and third trimester caffeine intakes were estimated by Wilcoxon test. Maternal and neonatal characteristics were expressed as the mean values with SDs in appropriate units or as percentages (%). Differences between caffeine intake groups were analyzed using ANOVA test for continuous variables and by Chi- square (χ^2^) test for categorical variables.

Logistic regression models were used to examine associations between maternal caffeine intake in categories and SGA. The results were expressed as odds ratios (ORs) with 95% confidence intervals (CIs) and *P*-values. Low-caffeine consumers were used as the reference group. Three regression models were used in the analyses. Model 1 was adjusted for maternal age and early pregnancy BMI. Model 2 was adjusted for model 1, smoking and energy intake. Model 3 was adjusted for model 2, the consumption of vegetables, fruits and berries and fish, and hypertension or pre-eclampsia during pregnancy. The association between the first trimester caffeine intake and SGA was also analyzed by dividing caffeine intake into quartiles, quintiles, deciles and into two groups based on the caffeine intake recommendation ($\le/>$200 mg). Also, coffee consumption was classified into tertiles and into two groups ($\le/>$100 ml), and the associations with SGA were examined by logistic regression. Pearson’s correlation was used to estimate the strength and the direction of the associations.

## Results

Table 1 presents the maternal caffeine intake and the sources of caffeine during the first and third trimester of pregnancy. Median-caffeine intake during the first trimester of pregnancy was 121 mg/day and increased to 134 mg/day in the third trimester of pregnancy. The EFSA recommendation for caffeine intake ($\le$200 mg/day) was exceeded by 32% of the study participants in the first and 38% in the last trimester of the pregnancy. Median coffee intake during the first trimester of pregnancy was 118 ml/day and in the last trimester 150 ml/day. Coffee was the main source of caffeine, covering 74% and 75% of caffeine intake during the first and the third trimester of pregnancy, respectively. In the first trimester, 44% and in the third trimester, 52% of coffee drinkers consumed caffeine more than recommended. The consumption of tea, cola and energy drinks, cocoa, and chocolate increased from the first to the third trimester of pregnancy. Only 4% of women in the first trimester and 2% in the third trimester consumed energy drinks. The consumption of energy drinks during pregnancy correlated with smoking, younger age, and shorter education (detailed data not shown).

**Table 1:** Maternal caffeine intake and the sources of caffeine during the first and third trimester of pregnancy in the Kuopio Birth Cohort (KuBiCo) Study

	First trimester intake		Third trimester intake		*P-*value^a^
	(*n* = 2007)		(*n* = 4362)		
	Mean	Median	Mean	Median	
	(Standard deviation, SD)	(Interquartile range, IQR)	(Standard deviation, SD)	(Interquartile range, IQR)	
Total caffeine (mg/d)	151.0 (137.5)	120.9 (272.4)	173.2 (135.4)	133.5 (256.2)	**0.001**
Coffee (ml/d)	158.8 (174.4)	117.9 (375.0)	185.0 (176.3)	150.0 (375.0)	**0.397**
Tea (ml/d)^2^	47.6 (57.7)	21.4 (54.3)	51.2 (3.7)	21.4 (54.3)	**0.007**
Cola drinks (ml/d)	59.1 (106.4)	33.3 (71.4)	74.9 (144.2)	33.3 (71.4)	** < 0.001**
Energy drinks (ml/d)^b^	78.3 (97.8)	33.3 (38.1)	81.5 (93.5)	33.3 (38.1)	** < 0.001**
Cocoa (ml/d)	15.4 (41.1)	10.0 (10.0)	17.1 (29.8)	10.0 (10.0)	** < 0.001**
Chocolate (g/d)	6.8 (7.2)	6.4 (3.4)	9.5 (9.6)	6.4 (16.3)	** < 0.001**

^a^*P-*values estimated by Wilcoxon test between the first and the third trimester intakes from those whom both food frequency questionnaires were available (*n* = 1262)

^b^Mean (SD) and median (IQR) values of consumers, not from all participants

During the first trimester of pregnancy, 67.9% of women drank coffee, while during the third trimester of pregnancy the corresponding rate was 67.4%. The correlation coefficient between coffee consumption in FFQ1 and FFQ2 was 0.93 (*P* < 0.001). In total, 3.3% of women did not drink coffee in the first trimester but drank it during late pregnancy. In contrast, 3.9% of women drank coffee in the first trimester but stopped drinking it towards the end of pregnancy. It seemed that coffee consumption is relatively constant in this study population during pregnancy.

Maternal and neonatal characteristics according to the three caffeine intake groups are presented in Table 2. In both the first and the third trimester of pregnancy, caffeine intake was higher the higher the maternal age, parity and the daily energy intake were. In the first trimester, the caffeine intake was greater in women with higher early pregnancy BMI, and in the third trimester, in those with greater gestational weight gain. Women in the highest caffeine intake group also smoked more in both the first and third trimester of pregnancy (21% and 19%, respectively).

**Table 2:** Maternal and neonatal characteristics among all participants and according to caffeine intake groups during the first and third trimester of pregnancy

Characteristic	First trimester intake				
	All	Low	Moderate	High	*P-*value^a^
	Participants	≤ 50 mg	51–200 mg	> 200 mg	
	(*n* = 2007)	(*n* = 655)	(*n* = 725)	(*n* = 627)	
Age (years)	31.2 (4.7)	30.5 (4.6)	31.3 (4.6)	31.9 (4.8)	** < 0.001**
Energy intake (kcal/d)	2046 (527)	1975 (541)	2039 (512)	2128.4 (527)	** < 0.001**
Body mass index (kg/m^2^)	25.3 (5.2)	25.2 (5.5)	25.0 (5.0)	25.8 (5.1)	**0.017**
Weight gain (kg)	13.6 (5.1)	13.5 (5.1)	13.4 (5.0)	13.9 (5.2)	0.215
Smoking (%)	11.3%	4.6%	8.7%	21.2%	** < 0.001**
Education > 15 y^b^	68.4%	70.1%	69.2%	65.4%	0.221
In a relationship (%)	96.7%	97.1%	96.4%	96.7%	0.771
Parity (%)					**0.004**
Nulliparous	55.0%	57.6%	57.0%	49.9%	
Primiparous	28.1%	28.2%	27.4%	28.7%	
Multiparous	16.9%	14.2%	15.6%	21.4%	
Vaginal delivery (%)	87.9%	90.0%	88.6%	84.9%	**0.013**
Newborn sex, boy (%)	50.0%	48.7%	49.2%	52.3%	0.377
Gestational age at delivery (day)	278 (11.4)	277 (11.9)	278 (10.7)	278 (11.6)	0.103
Preterm birth, < 37 w (%)	5.4%	6.9%	4.4%	5.1%	0.120
Birth weight (g)	3472 (498)	3475 (512)	3463 (484)	3480 (498)	0.806
Low birth weight, < 2500 g (%)	3.3%	3.8%	2.8%	3.5%	0.528
Macrosomia, > 4500 g (%)	1.6%	1.5%	1.2%	1.9%	0.606
Small for gestational age (%)	2.0%	0.92%	2.5%	2.6%	0.056
Large for gestational age (%)	1.8%	1.4%	2.4%	1.8%	0.385
Head circumference (cm)	34.9 (1.5)	34.9 (1.6)	34.8 (1.5)	34.9 (1.6)	0.770
Apgar Score 5 min	9.0 (0.82)	9.0 (0.79)	9.0 (0.70)	9.0 (0.99)	0.317
Duration of hospital stay (day)	3.3 (9.1)	3.3 (4.8)	3.7 (14.4)	3.0 (2.7)	0.362
Neonatal intensive care (%)	12.0%	13.3%	9.2%	13.7%	**0.018**
Congenital anomaly (%)	3.8%	3.4%	3.6%	4.6%	0.453

Third trimester intake				
All	Low	Moderate	High	*P-*value^a^
Participants	≤ 50 mg	51–200 mg	> 200 mg	
(*n* = 4362)	(*n* = 1069)	(*n* = 1623)	(*n* = 1670)	
31.1 (4.9)	30.1 (4.9)	31.2 (4.8)	31.6 (4.8)	** < 0.001**
2121.8 (525)	2037.2 (527)	2112.1 (529)	2185.3 (513)	** < 0.001**
25.2 (5.0)	25.3 (5.5)	25.0 (4.9)	25.3 (5.1)	0.248
13.5 (5.4)	13.5 (5.5)	13.2 (5.6)	13.8 (5.1)	**0.011**
12.7%	7.3%	10.4%	18.5%	** < 0.001**
66.2%	66.8%	67.7%	64.3%	0.195
96.3%	97.2%	95.9%	96.1%	0.184
				** < 0.001**
50.7%	59.1%	53.7%	42.4%	
30.8%	27.0%	30.5%	33.5%	
18.5%	13.9%	15.8%	24.1%	
87.6%	87.4%	87.4%	87.9%	0.888
50.4%	50.7%	49.7%	50.9%	0.756
278 (11.5)	277 (11.2)	278 (12.2)	278 (11.0)	0.085
5.1%	5.1%	6.0%	4.3%	0.067
3490 (516)	3494 (515)	3469 (541)	3507 (490)	0.108
3.6%	3.2%	4.1%	3.2%	0.287
1.6%	1.2%	1.7%	1.6%	0.563
2.4%	2.0%	2.7%	2.3%	0.456
1.8%	1.9%	1.7%	1.7%	0.955
35.0 (1.5)	35.0 (1.6)	34.9 (1.5)	35.0 (1.5)	0.640
9.0 (0.81)	9.0 (0.81)	9.0 (0.82)	9.0 (0.79)	0.419
3.2 (6.7)	3.2 (3.7)	3.2 (4.7)	3.1 (9.4)	0.871
11.4%	11.4%	11.6%	11.2%	0.941
3.9%	3.6%	3.3%	4.8%	0.061

^a^*P-*value estimated by Chi-square test for categorical variables and ANOVA for continuous variables. Values are means (standard deviations; SDs) or *n* (%)

^b^Education started in elementary school

Regression models for neonatal risk of being SGA according to maternal caffeine intake during the first and last trimester of pregnancy are presented in Table 3. In model 1, adjusted with the maternal age and early pregnancy BMI, the women who consumed moderate (OR 1.69; 95% CI 1.06, 2.69) or high (OR 1.45; 95% CI 1.05, 1.99) amounts of caffeine during the first trimester of pregnancy were more likely to have SGA newborns as compared to the women with low caffeine intake. The association remained significant after additional adjustment in Model 2 (adjusted with model 1, smoking and energy intake) (OR 1.66; 95% CI 1.04, 2.64 for moderate consumers; OR 1.39; 95% CI 1.01, 1.93 for high consumers) and in Model 3 (adjusted with model 2, the consumption of vegetables, fruits and berries and fish, and hypertension or pre-eclampsia) (OR 1.87; 95% CI 1.16, 3.02 for moderate consumers; OR 1.51; 95% CI 1.08, 2.10 for high consumers). The trend was similar in sensitivity analyses with different caffeine intake categorizations.

**Table 3:** The risk for being born small for gestational age newborn according to maternal caffeine intake during the first and third trimester of pregnancy

First trimester caffeine	Low intake ≤ 50 mg	Moderate intake 51–200 mg		High intake > 200 mg	
*n* = 2007	*n* = 655	*n* = 725		*n* = 627	
	OR (95% CI)^a^	OR (95% CI)	*P* value	OR (95% CI)	*P* value
**Small for gestational age**					
*Model 1*	1	1.69 (1.06, 2.69)	**0.028**	1.45 (1.05, 1.99)	**0.022**
*Model 2*	1	1.66 (1.04, 2.64)	**0.034**	1.39 (1.01, 1.93)	**0.046**
*Model 3*	1	1.87 (1.16, 3.02)	**0.011**	1.51 (1.08, 2.10)	**0.016**

Third trimester caffeine	Low intake ≤ 50 mg	Moderate intake 51–200 mg		High intake > 200 mg	
*n* = 4362	*n* = 1069	*n* = 1623		*n* = 1670	
	OR (95% CI)	OR (95% CI)	*P* value	OR (95% CI)	*P* value
**Small for gestational age**					
*Model 1*	1	1.18 (0.91, 1.54)	0.209	1.07 (0.89, 1.28)	0.468
*Model 2*	1	1.18 (0.90, 1.53)	0.228	1.05 (0.88, 1.27)	0.579
*Model 3*	1	1.18 (0.90, 1.53)	0.232	1.06 (0.88, 1.27)	0.549

Model 1 adjusted for maternal age and early pregnancy body mass index

Model 2 adjusted for model 1, smoking and energy intake

Model 3 adjusted for model 2, the consumption of vegetables, fruits and berries and fish and maternal hypertension or pre-eclampsia during pregnancy

^a^Values are odds ratios (95% confidence intervals) from logistic regressions

Regarding coffee consumption, the risk of SGA was highest among the women with the highest coffee consumption during the first trimester of pregnancy. According to the regression model 3, women in the highest tertile of coffee consumption (151–900 ml daily) had OR 2.44 (95% CI 1.03, 5.78; *P* = 0.043) as compared to participants in the lowest tertile with 0–10 ml daily consumption. When coffee consumption was divided into two groups and adjusted with Model 3, the consumption of more than 100 ml of coffee daily was likely to associate with the increased the risk of SGA compared to lower consumption, but the result was not statistically significant (OR 1.73; 95% CI 0.88, 3.42; *P* = 0.112). Caffeine intake and coffee consumption during the third trimester of pregnancy were not associated with SGA.

## Discussion

In this prospective birth cohort study in Finland, caffeine and its main source, coffee, were risk factors for SGA when the exposure was during the first trimester, but not when the exposure was during the third trimester. The risk of SGA increased considerably even when caffeine was used within EFSA recommendations ($\le$200 mg/day). Notably, the caffeine recommendations for pregnant women were exceeded by one-third of all women and half of coffee drinkers. The results can only be generalized to the Western population, where consumption of coffee is relatively high.

Our results are consistent with two earlier observational studies that have reported increased risks for SGA birth even with moderate caffeine intakes [21, 40]. However, in our study, the risk of SGA was much greater than found in these previous studies. Similarly to our study, Hoyt and associates [41] reported 57% increased risk for SGA birth with higher caffeine use (> 300 mg/day) in their population-based case–control study. According to the meta-analysis including 15 SGA studies, it was stated that each additional 100 mg daily amount of caffeine increase the risk of SGA by 10% [10]. In our study, dose response was not observed, rather the risk was highest with the moderate intake.

### SGA

In our study, the risk of having an SGA newborn was 2% of all participants and 2.5–2.6% among women who consumed caffeine moderate or high amounts. Although the prevalence of SGA children born in this study population was low, a significantly higher prevalence was observed in the group of those who consumed the most caffeine (2.6%, *n* = 16/627) as compared to those in the lowest intake group (0.9%, *n* = 6/655) in the first trimester. The prevalence of SGA was at the similar level as in a Norwegian cohort study [21] and in an earlier Finnish birth cohort study [42]. The definition of SGA varies, and in some countries and studies, SGA has typically been defined as a newborn who is below the 10th percentile of birth weight for gestational age [17]. In our study, the definition of SGA was defined as having a birth weight more than two SDs below the mean weight for the gestational age which is stricter and gender and region-based way to select the cases. Compared to birth weight alone, SGA is more versatile concept for describing restricted fetal growth. In addition to SGA as a neonatal outcome, SGA newborns are in the risk for several health problems later in life.

In the analyses of this study, the associations between caffeine intake and SGA were strengthened when adjusted regression models were used. The association were most pronounced in the model with SGA-related covariates such as dietary factors and hypertension or pre-eclampsia. It is well known that caffeine intake is associated with unhealthy lifestyle habits, such as smoking [5, 43] which is also the known risk factor for SGA. Thus, we also adjusted the statistical analyses by smoking.

Exposures in early pregnancy may be more relevant for SGA because early pregnancy is an essential time for the development of the placenta and fetus. The placenta starts to develop in the very early pregnancy and maternal lifestyle can detrimentally influence early placental development and its function later [44] and contribute to restricted fetal growth [45]. Caffeine exposure during a sensitive time frame of pregnancy may also induce epigenetic changes in the developing fetus [46]. It is possible that the consumption of caffeine later in pregnancy does not affect fetal growth if the placenta has developed normally in the early pregnancy.

### Caffeine intake during pregnancy

In Finland, coffee intake is the highest worldwide [47]. The Finnish recommendations for pregnant women state that caffeine should not be consumed more than 200 mg/day and energy drinks are not recommended during pregnancy at all [48]. Based on our recent and previous studies [5], pregnant women consume caffeine a lot. In this study, median-caffeine intake was 121 mg/day in early pregnancy and even higher, 134 mg/day in late pregnancy. In comparison to one recent Nordic study [21], maternal caffeine intake was much lower: median 58 mg/day during mid-pregnancy. The wide distribution of caffeine intake increases the reliability of our study. We also evaluated all dietary sources of caffeine, not only coffee. However, coffee was the primary source of caffeine covering three quarters of intake. Because the consumption of energy drinks was low during pregnancy (2–4% of pregnant women), it was not possible to assess its independent association with SGA.

According to our results, caffeine intake from various sources increases from the first to third trimester of pregnancy. We found that total caffeine intake during the first, but not the third trimester, was significantly associated with the increased risk of SGA. Because our FFQ covered the three preceding months, the first trimester questionnaire extended to the pre-pregnancy period in some participants, and since the first trimester FFQ was closed already at the end of the 13th week of pregnancy, not all study subjects completed the first nutrition questionnaire. Thus, if the results of our study were repeated in other studies, it would be important to extend caffeine recommendations also for women who are planning for pregnancy.

Nutrition studies have some limitations, such as self-reporting and recall bias related to the dietary intake methods. In caffeine-containing drinks, factors like the brand, the type of beans and leaves, preparation, and the amount of caffeine may also cause bias, considering our approximation of maternal caffeine intake [49]. However, Gleason et al. [9] found that the results from self-reported and biomarker-measured caffeine exposure are consistent with each other. As a dietary assessment method, the FFQ is reliable for epidemiological cohort studies like KuBiCo [50]. In this study, nutritional factors were assessed by using the validated and pregnancy-modified FFQ. The presented factors and limitations were addressed by using relevant statistical analyses with adjustments for several potential confounders. The strengths of this study were the population-based prospective study design and large range in caffeine intake due to the high consumption in Finland. Also, careful adjusting with potential cofounding factors brought novelty value to the research.

## Conclusions

We found that moderate to high maternal caffeine intake during the first trimester of pregnancy was associated with the higher risk of delivering a SGA baby. Thus, even caffeine intake within the current recommendations (< 200 mg/day) may not appear to be safe for fetus. Because caffeine intake was exceeded in one third of all study women, and close to half of the coffee drinkers during the early pregnancy, recommendations for caffeine and coffee intake should be given to women already planning pregnancy. Our results also indicate that caffeine intake during early pregnancy is more significant for the development of SGA than caffeine intake later in pregnancy. Future studies should address more periconceptional factors and their importance in the placenta-related outcomes such as SGA.
